# Teaching NICO How to Grasp: An Empirical Study on Crossmodal Social Interaction as a Key Factor for Robots Learning From Humans

**DOI:** 10.3389/fnbot.2020.00028

**Published:** 2020-06-09

**Authors:** Matthias Kerzel, Theresa Pekarek-Rosin, Erik Strahl, Stefan Heinrich, Stefan Wermter

**Affiliations:** Knowledge Technology, Department of Informatics, University of Hamburg, Hamburg, Germany

**Keywords:** crossmodal learning, developmental robotics, neurocognitive models, human-robot interaction, visuomotor learning

## Abstract

To overcome novel challenges in complex domestic environments, humanoid robots can learn from human teachers. We propose that the capability for social interaction should be a key factor in this teaching process and benefits both the subjective experience of the human user and the learning process itself. To support our hypothesis, we present a Human-Robot Interaction study on human-assisted visuomotor learning with the robot NICO, the Neuro-Inspired COmpanion, a child-sized humanoid. NICO is a flexible, social platform with sensing and manipulation abilities. We give a detailed description of NICO's design and a comprehensive overview of studies that use or evaluate NICO. To engage in social interaction, NICO can express stylized facial expressions and utter speech via an Embodied Dialogue System. NICO is characterized in particular by combining these social interaction capabilities with the abilities for human-like object manipulation and crossmodal perception. In the presented study, NICO acquires visuomotor grasping skills by interacting with its environment. In contrast to methods like motor babbling, the learning process is, in part, supported by a human teacher. To begin the learning process, an object is placed into NICO's hand, and if this object is accidentally dropped, the human assistant has to recover it. The study is conducted with 24 participants with little or no prior experience with robots. In the *robot-guided* experimental condition, assistance is actively requested by NICO via the Embodied Dialogue System. In the *human-guided* condition, instructions are given by a human experimenter, while NICO remains silent. Evaluation using established questionnaires like Godspeed, Mind Perception, and Uncanny Valley Indices, along with a structured interview and video analysis of the interaction, show that the robot's active requests for assistance foster the participant's engagement and benefit the learning process. This result supports the hypothesis that the ability for social interaction is a key factor for companion robots that learn with the help of non-expert teachers, as these robots become capable of communicating active requests or questions that are vital to their learning process. We also show how the design of NICO both enables and is driven by this approach.

## 1. Introduction

In the future, robots may perform complex visuomotor tasks in domestic environments as human assistants and companions. Today, this is still a challenge due to the complexity of the dynamic, non-standardized environments and tasks involved. A promising approach for coping with this complexity is to take inspiration from biological systems and develop neurocognitive learning models embodied in developmental robots (Cangelosi and Schlesinger, 2015) that learn, similar to a human child or infant, from interaction with the environment and imitation of, or teaching by, adult experts. A spectrum of such learning approaches exists in the literature, ranging from relying entirely on the imitation of a human teacher to nearly autodidactic approaches without any human assistance. Imitation approaches often face challenges when the robotic anatomy diverges from that of the human demonstrator: though anthropomorphically designed, robotic hands usually do not match the degrees of freedom (DoF) of the human hand sufficiently to allow a direct mapping (Gupta et al., 2016). Furthermore, external tracking approaches for hands and objects are often constrained to laboratory settings. On the other hand, deep reinforcement learning promises human-level control (Mnih et al., 2015) through autonomous interaction with the environment. The agent learns through trial and error to achieve a given goal. However, most robot platforms and environments are not suited to the large number of interactions in the real world or the possibility of harmful actions. Therefore, many intermediate approaches have been developed that combine autonomous learning with human expert knowledge in the form of instructions (Cruz et al., 2016), or imitation (Gupta et al., 2016). The presented research follows the concept of developmental robotics, which aims to leverage efficient learning strategies inspired by nature. We adopt the principle of scaffolding, a teaching approach based on collaborative interaction between the learner and an expert (Newson, 1979), which plays a crucial role in early human development, for a robot.

We hypothesize that there are two requirements of the robotic learner to enable successful scaffolding:**(1) Sensory and motoric similarity:** human and robot need to have a substantial overlap in their motor and sensory abilities to enable the robot to profit from human demonstration and to enable the human to affect the learning of the robot positively. Especially, non-expert users rely on their intuitive ability for human-to-human teaching to convey their skills. Different sensory modalities, body forms, and degrees of freedom can hinder this transfer. Therefore, a robotic companion needs to mimic human sensory and motor abilities to a certain degree. As an example, the way a human grasps or handles an object might not be applicable to a robot with a non-hand-like end-effector. Also, the robot's size is essential; while smaller robots might be easier to construct and require less powerful motors or materials, a robot must have a sufficient size to operate efficiently in a domestic environment.**(2) Approachability and social interaction:** the robot's physical design and behavior need to encourage users to engage in teaching interactions. Not only are safety issues a concern when it comes to physical human-robot interactions; perceived safety and approachability are important because they encourage especially non-expert users to engage in (physical) interactions to improve the learning outcome, for example, reaching into the robot's workspace while the robot is performing a manual task. Furthermore, the robot should encourage an intuitive, natural teaching interaction that relies on natural language and social cues.

**Figure 1:**
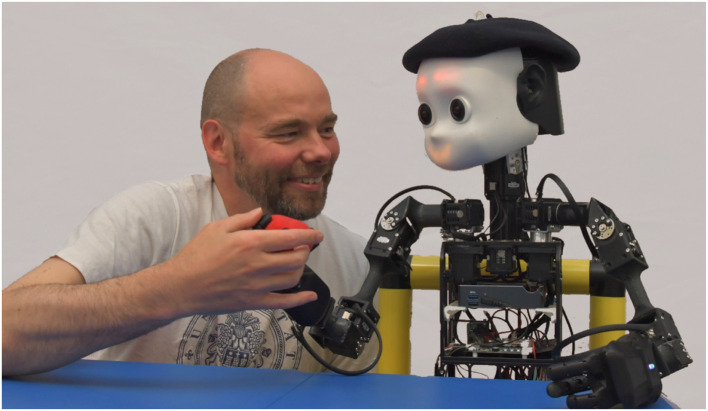
NICO, the Neuro-Inspired COmpanion, is being taught how to grasp a training object by a human assistant and is giving positive feedback with its facial emotion display.

Through meeting these criteria, we expect the Neuro-Inspired COmpanion robot, NICO (see Figure 1), an open-source developmental robot platform developed by the Knowledge Technology group^1^, to be able to acquire visuomotor skills with the assistance of non-expert users. We present an update to the NICO platform with a focus on the properties that are relevant for this study and a review of related studies; we examine the assumption that social interaction and human-like sensorimotor abilities are a key to robots learning from humans by conducting a Human-Robot Interaction study with 24 participants in which we evaluate the effect of an active role of a humanoid in a grasp-learning experiment. In a novel comparative crossmodal, visuomotor learning study, NICO is supported by a non-expert participant in a visuomotor learning task. This study, for the first time, evaluates the interplay between NICO's social interaction and visuomotor learning abilities. NICO learns to grasp by repeatedly placing and re-grasping an object at different positions in its workspace. During this semi-autonomous grasp learning, NICO requires the aid of human assistants to initialize the learning process and to provide aid in case NICO loses the object. Two experimental conditions are evaluated, in which NICO either takes a passive or an active role in the learning process: in the baseline *human-guided* condition, all instructions toward the participant are given by the experimenter; in the *active learning* condition, the robot uses a crossmodal Embodied Dialogue System to actively guide a non-expert participant through the learning process and to request assistance when needed. The experimenter is present during this time but does not communicate with the participant. We show that the active, communicative, and emotional engagement of the robot in a teaching situation leads not only to a subjectively better rating of the robot using a set of established measures for HRI research but also to an increase in the engagement of the human, non-expert teachers, which in turn can lead to better visuomotor learning results.

We would also like to address the methodological gap between *machine learning* in robotics and *neurorobotics*. The embodiment of state-of-the-art machine neural machine-learning in a physical platform allows training and evaluation that is hardly possible in simulation, e.g., physical interaction between a robot's hand and a soft, deformable object. More importantly, we argue that research communities for machine learning in robotics and developmental robotics are growing closer together. While classical roboticists focus on *human-in-the-loop* approaches that rely on imitation learning and demonstration, developmental roboticists have been researching scaffolding by caregivers to learn complex cognitive and visuomotor skills. The underlying idea is the same: leveraging human competence can be an essential part of robotic learning. This competence can be supplied by trained experts as well as non-expert users. In the latter case, one of the main goals is to enable these non-expert users to use their intuitive teaching abilities in a robotic scenario, which in turn relies on an intuitive and natural communication with the robot.

Our main claim is that non-expert users can teach visuomotor skills to a developmental robot; however, the more these non-experts are engaged in the teaching experience, the more they tend to use intuitive teaching approaches that in the end lead to more efficient teaching. This effect requires a humanoid platform that enables intuitive and engaging social interaction and, at the same time, has sufficient sensing and motor abilities for the learned action. In section 2, we report on different robot platforms and robotic visuomotor learning approaches. In section 3.1, we present the updated NICO and a comprehensive review of studies on its sensory, motor, and HRI abilities. We show how its design both enables and is driven by the interplay of social interaction and sensorimotor learning by summarizing previous studies that often focused either on social interaction or on crossmodal and visuomotor learning. We bring these aspects together in section 4, where we detail the grasp-learning approach, the Embodied Dialogue System, and the setup for the HRI experiment, which couples social interaction and visuomotor learning, and we show, in section 5, how an engaging social interaction can enhance the quality of robotic visuomotor learning. We conclude with a discussion of the results and examine their implications as a contribution to the future development of learning companion robots in section 6, finding that the ability of a balanced robotic platform to engage non-expert users can benefit the learning of non-social tasks. The social aspect not only enhances the user's subjective experience but also to enables non-experts to apply intuitive teaching approaches.

## 2. Related Work

### 2.1. Humanoid Platforms

Today, a wide range of robots is available, though not all of them fulfill the above-mentioned criteria of possessing a sensory and motoric similarity to humans in addition to an approachable design and social interaction abilities: a humanoid is expected to have two arms with a human-like range of motion and hand-like end-effectors to use tools and manipulate objects in domestic environments. Often, the hands' fingers have tactile sensors to enhance grasping, tool use, and in-hand manipulation but also to create shared, embodied sensory concepts with human interaction partners regarding haptic properties like softness or texture. The locomotion of a humanoid is usually bipedal. Though complex to realize, walking allows the navigation of domestic environments, for instance, a cluttered floor. However, for better stability and easier handling, many platforms use a wheeled base instead. A humanoid also has a head with eyes. Though other sensing setups might be more efficient for specialized tasks, such as 360° laser scanners for mapping, eye-like cameras can enable shared attention with human interaction partners and thus also fulfill a critical communicative role. Many humanoids feature some form of emotional expression on their face, ranging from color changes of status LEDs to stylized and animated facial expressions and mouth movements. An alternative to an actual face is a monitor or tablet that displays a virtual avatar or face. Another important criterion for research platforms is an open design that allows customization of the platform toward novel experimental setups, easy maintainability of the platform, and compatibility with common software standards.

One way to categorize humanoids is by their size. Small, infant-sized humanoids are affordable, easy to handle, and secure. For example, the NAO from Aldebaran is well-used in research on developmental robots, while the DARwIn-OP from Robotis was a popular walking platform for the RoboCup competitions (Ha et al., 2011). However, these small platforms cannot interact with most domestic environments, cannot use tools, and are not able to manipulate everyday objects.

Child-sized robots overcome this challenge while still being relatively easy to handle in terms of weight and size. The iCub resembles a 3.5-years-old child (Metta et al., 2010). The iCub has many relevant features for developmental robot research and HRI: 53 human-like DoF, five-fingered hands, eyes with mechanical gaze shift, an LED-based display for stylized emotion expression, and optional tactile sensing skin. However, its holistic design impedes individual modifications. More modular are the NimbRo-OP (Schwarz et al., 2013) and its slightly larger, novel design Nimbro-OP2X (Ficht et al., 2018) from the AIS (Autonomous Intelligent Agents) group of the University of Bonn. These platforms are designed for the RoboCup TeenSize and AdultSize league and, as such, prioritize walking over manipulation ability: the arms have non-actuated end-effectors and serve primarily for balance and getting up from a prone position. The Poppy robot (Lapeyre et al., 2014) is a 3D-printable open-source robot developed in 2014 by a research group at the French Institute for Research in Computer Science and Automation (INRIA). The objective of the robot is to be a robot base for scientists, students, and artists originally aiming to study the role of morphology in sensorimotor control. The software API of the Poppy robot is based on Pypot^2^, a framework for modeling controllers for custom robots, which is used by the NICO robot as well. The Reachy robot is a commercial robot torso developed by Pollen robotics^3^ in 2017. The robot has 7-DOF arms and can lift up to 500 g (Mick et al., 2019). The software of Reachy is Python-based. The strengths of the robot seem to be in the field of manipulation, as the capabilities of the arm are sophisticated for a robot of this size category. The Pepper by Softbank (formerly Aldebaran) (Pandey and Gelin, 2018) is mainly designed for Human-Robot Interaction. Its human-like torso is fitted onto a wheeled platform. Pepper has 20 DoF and human-like arms with five-fingered hands; however, its arms and fingers are mainly designed as a means for making gestures.

Soft-skin platforms offer a more realistic human-like appearance. The CB2 (Child with Biomimetic Body) from Osaka University (Minato et al., 2007) is a 130-cm tall platform for cognitive developmental robotics and features soft skin and flexible pneumatic actuators; it has a total of 63 degrees of freedom. Its face has actuators for eyeballs, eyelids, eyebrows, cheeks, and mouth to display emotions. In addition to cameras and microphones, skin tactile sensors in the skin can mediate haptic interaction. It is designed with a view to social interaction with a human caregiver. Affetto (Ishihara and Asada, 2015) has a similar design. It is an upper-body platform that has the proportions of an 80-cm tall child and has 22 degrees of freedom. It is designed to appear human-like, including in terms of its visual and tactile impression. Among the adult-sized robots, the high-performance biped Talos from PAL robotics (Stasse et al., 2017) is a further development of their REEM robot and offers a platform for research in complex industrial environments. It is well-suited for physical manipulation tasks and can traverse rough terrain but is not designed for social interaction. The PR2 from Willow Garage, a wheeled robot with two 7-DoF arms endowed with grippers with tactile sensors, has a similar function. Like the Talos, it has no means for emotion-expression and is instead designed for physical tasks rather than HRI. Finally, the InMoov (Langevin, 2014) is an open, 3D-printable robot with a human-like design and tendon-operated five-fingered hands. Instead of displaying emotions, it can move its jaw to emulate talking.

In summary, many robotic platforms are available, though currently, no single platform offers a combination of object manipulation, sensing, and HRI qualities in an affordable and open design. This gap in the state of the art is addressed with the NICO robot (Kerzel et al., 2017c), whose design will be summarized below.

### 2.2. End-to-End Visuomotor Learning

Visuomotor skills map raw sensory input to motor actions. Modular approaches divide this task; they process sensory information into explicit internal representations like coordinates that are then used as input for modules like inverse kinematics solvers. However, these approaches often have difficulties adjusting to novel challenges due to their lack of inherent learning ability. A complementary approach is to learn visuomotor skills through interaction with the environment (Cangelosi and Schlesinger, 2015). Deep reinforcement approaches employ trial and error learning. Based on initial random exploration, rewards for successful actions drive the learning of visuomotor policies. Lillicrap et al. (2016) introduced the Deep Deterministic Policy Gradient algorithm (DDPG) to solve a series of visuomotor tasks in a simulated two-dimensional environment. This approach is based on early direct motor model learning, where motor skills are learned in the target space only based on minimizing the error from observations Rolf et al. (2009); Nguyen-Tuong and Peters (2011). However, adapting these algorithms to physical robots is challenging. The trial and error exploration can be harmful to the robot or its environment; a large number of required trials can cause material stress and might be too time-consuming. Therefore, extensions to the DDPG algorithm and related algorithms have been suggested to enhance the sample efficiency and reduce the required training episodes. These approaches leverage the principles of intrinsic curiosity (Hafez et al., 2019), imagination (Andrychowicz et al., 2017), and task simplification (Kerzel et al., 2018). However, the basic problem of reinforcement learning of possibly unproductive and harmful exploratory actions remains.

This issue can be addressed in several ways. Nair et al. (2018) combine imitation and reinforcement learning. Instead of random explorations, the learner first learns to mimic the actions of a human teacher. The learner then refines its policy for exploration once a sufficient level of performance is reached to avoid unproductive or harmful actions. The basic idea behind this approach is to give the learner a set of *good* samples to bootstrap the learning process. A variation of this strategy is not to use an external teacher to imitate but to design the learning setup such in a way that the learner can generate these *good* learning samples autonomously. In the Hindsight Experience Replay (HER) (Andrychowicz et al., 2017), imagination is used after the execution of an action to, in hindsight, imagine the optimal goal for the previously executed action. This imagined training sample is then used to update neural policy models. However, though the creation of imagined samples works well in simulated environments, it can prove difficult in real environments. A related strategy is to let the learner generate *good* samples through physical actions. This strategy is employed by Levine et al. (2016), who utilize the known forward kinematic of the PR2 robot. Samples are generated by having one of the robot's hands move a target object while the other hand tries to grasp the object. Kerzel and Wermter (2017b) introduced a related approach where a robot generates samples for grasp learning by repeatedly placing and re-grasping an object at a random location. This approach has the advantage that the kinematics of the robot do not need to be known. However, human assistance is needed to initialize the process and to interfere in the case of re-grasping errors. A second approach, adopting a strategy from human learning, is to have an expert observe the reinforcement learning process and interfere in critical situations by giving advice and warnings to the learner in case of harmful actions (Cruz et al., 2018a). In summary, human teachers play an essential role in making reinforcement learning more sample-efficient, be it as models for imitation, physical assistants, or advisers.

### 2.3. Natural Teaching of Robot Learners

To ease the transfer of humanoid social robots from laboratories to the cluttered surroundings of domestic life, they need to be able to adapt their behavior dynamically and learn new skills through the instructions of non-expert human users. Humanoid social robots have the advantage that they generally foster a human-like interaction with the user, allowing users to easily anthropomorphize the artificial agent (Epley et al., 2007). Social interaction through spoken dialogue is the most intuitive way to enable such communication since it does not require additional knowledge and training from the non-expert user. The robot has to be a transparent learner, with its observable behavior and spoken feedback motivating the user to teach it further.

In a study by Thomaz et al. (2006) examining the way people teach a virtual agent in a reinforcement learning simulation, evidence was found for people's willingness to view their interaction and teaching of the agent as a collaboration. The human teacher guides and adjusts the training behavior of the agent, with a tendency toward positive feedback. Even without any specific amplifying behavior by the artificial agent, there seems to exist a clear concept of partnership in human-robot teaching scenarios. However, in comparison to a virtual agent, a physical robot has to be much more transparent about its intentions and internal states to ease the cooperation between human and robot.

A typical teaching cycle usually consists of the teacher demonstrating the desired skill for the student, followed by a series of supervised repetitions by the student. During these repetitions, the teacher might offer spoken feedback, display corrective behavior, or provide additional demonstrations to further improve the performance of the student (Nicolescu and Mataric, 2003). To enable teaching behavior that feels natural to the teacher while being effective for the robot learner, one must consider the design and behavior of the artificial agent. A childlike design, according to the baby schema (Lorenz, 1943), with round eyes set low in a comparatively big head, can help in facilitating intrinsic teaching methods like scaffolding.

Scaffolding is a form of assistive teaching regularly and often unknowingly displayed by human adults when interacting with children or infants (Breazeal, 2002). While infants are not capable of actually requesting assistance, they display a form of proto-social response that resembles an adult's behavior closely enough that the caregiver can assign meaning to them and act accordingly. By reinforcing the infant's interaction with the environment, the caregiver can encourage and assist the learning of new abilities (Newson, 1979). The adult handles the parts that are beyond the infant's or, in our case, the robot's capabilities, allowing them to focus on solving the simpler parts of the problem first. The learning process is supported by the adult giving affective feedback, reducing distractions, and simplifying the problem in a way that allows the learner to recognize the solution to a problem before being able to implement it (Breazeal, 2002).

Designing the robot as an approachable, transparent interaction partner allows the human-robot team to show better performance and the learner to reach a higher level of competence. In a study by Srinivasan and Takayama (2016) examining how the behavior of the robot during the interaction influences people's willingness to help it, one seemingly obvious conclusion could be drawn: robots that get assistance from people tend to accomplish more.

## 3. NICO, The Neuro-Inspired COmpanion

### 3.1. NICO Robot Platform

To create a robotic research platform for embodied neurocognitive models based on human-like sensory and motor capabilities that is at the same time well-suited for HRI studies, the Knowledge Technology group at the University of Hamburg designed the NICO humanoid (Kerzel et al., 2017c)^4^.

**Figure 2:**
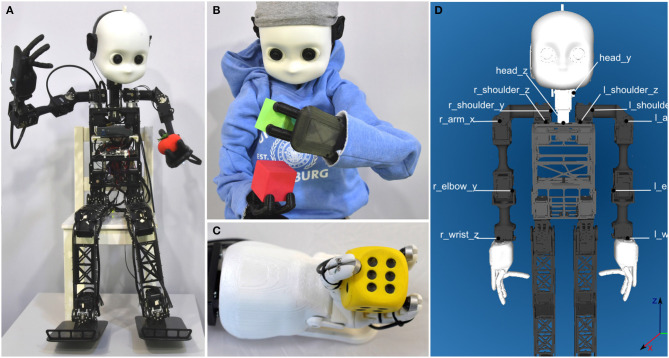
**(A)** NICO humanoid robot sitting on child-sized furniture. **(B)** NICO can wear regular clothing without being hindered in its motor abilities. **(C)** NICO's three-fingered hand with tactile sensors. **(D)** Schematic depiction of the mechanical design of NICO's upper body.

The first version of NICO was developed based on the NimbRo-OP, which was discussed in section 2.1. It is constructed mainly from 3D-printed parts and Robotis Dynamixel servomotors, endowing it with simple maintenance and high flexibility. This flexibility was used to gradually improve NICO, driven by experience from experimental setups and research. The designers followed a modular approach: each new functionality of the robot was first evaluated and iteratively improved before it was integrated with other functionalities. Following this scheme, a description of the sensory, motor, and HRI capabilities of NICO are given below alongside a review of scientific studies where these capabilities have been used. Figure 2 shows NICO with optional clothing and a close-up of its robotic hand with embedded tactile sensors.

#### 3.1.1. Physical Form and Appearance

NICO stands 101 cm tall and has a weight of 7 kg, with its body proportions and degrees of freedom resembling those of a child between the ages of three to four. NICO's face is adapted from the open iCub design, giving it a stylized, child-like appearance. In its standard design, NICO has no outer shell, i.e., it is possible to see through the frame of the robot. To alleviate this, a 3D-printed cover is being developed. Furthermore, its child-like anatomy allows the robot to wear off-the-shelf clothing.

#### 3.1.2. Motor Capabilities

NICO has 30 DoF, which are distributed as follows. Two DoF perform yaw and pitch movements of the head, which has an important signaling function in human-robot interaction, in addition to supporting joint attention and addressing communication partners. The arms have 6 DoF, with the shoulder forming a cluster of three motors that mimic the physiology of the human shoulder ball joint. An additional DoF allows bending of the elbows, and the final two DoF for wrist rotation and wrist flexion are provided by the Seed Robotics SR-DH4D articulated hands. These three-fingered hands are tendon operated; two motors contract the two linked index fingers and the opposed thumb. The tendon operation emulates hand synergies during grasping (Mason et al., 2001) to simplify the control during this complex process: only two DoF for closing the hand can securely grasp a wide range of different objects. Figure 2D shows a schematic depiction of the mechanical design of NICO's upper body. For locomotion, each of NICO's legs has three DoF in the hip joint, one DoF in the knee, and two DoF in the foot.

#### 3.1.3. Sensory Capabilities

NICO's head features two parallel See3CAM CU135 cameras with 4K resolution (4,096 × 2,160). The cameras have an opening angle of 202°. Via the API, the camera can be configured to transmit only parts of the image and thus constrain the field of view to a human opening angle of 70°. This results in a reduced amount of data and the possibility of realizing virtual gaze shifts. NICO's head is endowed with two Soundman OKM II binaural microphones embedded in realistically shaped and 3D-printed pinnae, which allows human-like binaural hearing for vertical and horizontal sound source localization. The location of the microphones and the dampening factor of the head and also of the pinnae have been designed to mimic human-child anatomy for providing a realistic distortion of the sounds. To reduce ego-noise and improve speech recognition, NICO's head was designed without internal fans, mechanics, or motors. Haptic sensing subsumes proprioception and tactile sensing. While proprioception provides information about body posture, movement, and forces, the tactile modality registers deformation, vibration, and temperature. Both sub-modalities are realized in NICO: information about motor position and torque provide a proprioceptive sense for all DoF. To allow faster and more precise measurement of forces in motors even during movements and under load, the energy supply to all motors has been redesigned to exclude artifacts from power spikes due to energy-intensive motions. For tactile sensing, OPTOFORCE OMD-10-SE-10N7 force sensors were installed in all three fingertips of each hand. These dome-shaped sensors are slightly deformable and measure forces of up to 10 N in three dimensions at up to 400 Hz, making them well-suited to picking up vibration.

#### 3.1.4. Interaction Capabilities

**Figure 3:**
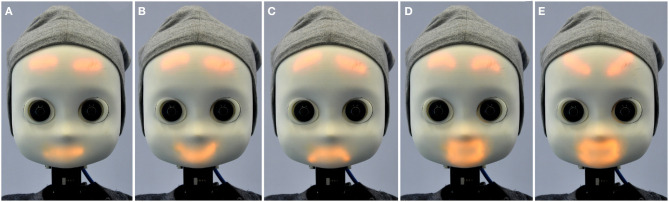
NICO's facial emotion display showing different expressions: **(A)** neutral, **(B)** happiness, **(C)** sadness, **(D)** surprise, and **(E)** anger.

NICO's head is fitted with three LED arrays in the mouth and eye areas that can display stylized facial expressions. The areas behind the eyes consist of 8 × 8 LEDs; the array in the mouth area consists of 16 × 8 LEDs. The thickness of the 3D-printed head is reduced in the respective areas and optimized to allow the LEDs to shine through the material while blurring individual lights. A set of fixed emotions can be displayed, as well as freely programmable patterns; thus, emotional expressions can also be learned over time or be adjusted to individual interaction partners. Figure 3 shows examples of expressions for happiness, sadness, surprise, anger, and a neutral mood. These facial displays can give intuitive feedback to the user about the state of NICO in the context of a task. In addition to this specialized facial display, NICO, like many robotic platforms, has an internal speaker for uttering spoken messages and can express non-verbal social cues like gestures, poses, and head movements.

#### 3.1.5. API and Virtual Model

The NICO API supports direct control via Python and the robot operating system ROS. Python allows easy integration into the most common frameworks for GPU processing and deep learning like Tensorflow (Abadi et al., 2016). This gives scientists an easy way to embody neurocognitive models into the robot. NICO's full functionality can also be accessed via ROS (Quigley et al., 2009), the de facto standard in the robotics community, to allow easy sharing of software modules. The low-level motor control of the API is based on PyPot (Lapeyre et al., 2014), which was extended to support NICO's hands. The use of Python makes it possible to utilize existing libraries for control and preprocessing of sensory information, such as OpenCV for camera and PyAudio for microphone recordings. A set of predefined facial expressions is provided for the Arduino-controlled facial emotion display. As virtual environments are often used in robotics research for allowing extended and controlled experiments without strain to the robotic hardware, a virtual realization of NICO for the V-REP robotics simulator (Rohmer et al., 2013) is provided. V-REP supports simulated physical interactions, including forces and friction between different objects. Additionally, the robot model is provided in the established Unified Robot Description Format (URDF) for use in other simulation environments. The URDF description contains information about the kinematics of the robot, its collision model, and visual representation. The API allows seamless switching between real and simulated environments.

### 3.2. NICO Evaluation and Studies

Following the design strategy to iteratively evaluate and improve each functionality of the robot before integrating it in larger experiments, a set of studies has been conducted involving NICO's motor, sensory, and human-robot interaction capabilities^5^. In some studies, the main scientific focus was not on the robot itself but on the neurocognitive models embodied within it. However, these studies are especially valuable for NICO's ongoing design process, as they provide feedback under realistic research conditions.

#### 3.2.1. Embodied Sensing Evaluation

***Embodied Visual Perception*. **Compared to the typical applications of computer vision approaches, there are no differences in a robotic vision system. Therefore, the performance of state-of-the-art approaches for object detection, e.g., RetinaNet (Lin et al., 2017) can be utilized without limitations on NICO. However, a robot offers the ability to combine vision with active object manipulation: the robot can move and turn an object to learn a more elaborate visual representation. Additionally, object manipulation can also be used to train and evaluate models for object tracking under deformation and occlusion. Heinrich et al. (2019) recorded the *NICO-object interaction dataset* featuring sixty object-hand interactions, including different push, pull, grasp, and lift actions on a broad range of toy objects that show diverse behaviors, such as rolling, bouncing, or deformation. The exploration procedures were inspired by typical child-like behavior that could be realized on a humanoid. The dataset was used to evaluate the HIOB framework, an adaptive convolutional object tracker based on an incremental update mechanism. Josifovski et al. (2018) applied a convolutional neural network for object detection and pose estimation trained with 3D-models of NICO's hand. Though the pose of the hand can be computed via forward kinematics, such models can contribute to developmental approaches in which the kinematics of the robot are learned. Furthermore, the work demonstrates the transfer of models trained on the simulated model to the real world.

***Embodied Audio Perception***. Like computer vision, audio processing on a robotic platform does not differ greatly from any non-robotic audio task. However, the robot's ego-noise during operation and its ability to actively manipulate objects to elicit audio information have to be considered. Humanoids can perform common audio exploratory procedures like shaking an opaque container to gain insight into its content. Eppe et al. (2018) and Strahl et al. (2018) recorded an audio dataset with 1,080 samples of active audio exploration of 30 capsules filled with different materials, which NICO could shake with its hand, to train a recurrent neural network classifier. High classification accuracy of 91% was achieved due to the low ego-noise of the robot in the head area.

***Embodied Haptic Perception***. In contrast to audio and vision approaches, haptic perception is inherently based on active exploration to gain information about handled objects and materials. Compressing or squeezing an object can give information about the compliance of the material, while forces along the movement direction during lateral motions reveal static and slip friction as well as texture information. NICO's haptic sensory setup enables the use of human-like haptic exploratory procedures: in two studies, the use of lateral motion across surfaces to gain texture information and the use of squeezing objects to gain information about their compliance and shape was evaluated. Kerzel et al. (2017a) collected a 3200-sample dataset of lateral motions over a set of 32 samples of common household materials ranging from metal to different fabrics or cardboard. High classification accuracy of 99% could be achieved with a neural model. The study could also positively evaluate the robustness of the sensors; in over 5,000 trials, no wear and tear to the sensor occurred. Kerzel et al. (2019b) collected a dataset of human-inspired active haptic exploration of 16 different toys by enclosing and squeezing the objects in the robot's hand. These objects range from foam dice to different plush and plastic figures. The dataset contains 100 active exploration trials for each of the 16 objects; in each trial, seven haptic sensory channels were recorded for 52 time steps. A neural model that integrates the different haptic sensory channels over time achieved a 66.6% classification accuracy. Both studies showed that a key to recognizing haptic properties is the integration of motoric, proprioceptive, and tactile information. To this end, the NICO API is designed to synchronize motor commands and different sensory streams.

#### 3.2.2. Motor Learning Evaluation

Several approaches for grasp learning have been evaluated on NICO: Hafez et al. (2017, 2019) successfully evaluated curiosity-driven reinforcement learning both on a simulated and on a physical NICO. For the physical experiments, full training of the deep RL approach was conducted without human supervision for over 50 h during which NICO performed arm movements and grasp actions. This uptime attests to the robustness of NICO's hardware. Cruz et al. (2016, 2018a,b) used a virtual model of NICO to develop and evaluate interactive reinforcement learning by allowing NICO to receive parent-like advice during a simulated cleaning task. Vocal commands and hand gestures were supplied during training and could be shown to enhance the training efficiency. These studies show the effectiveness of intuitive supervision by non-expert users during domestic tasks that are enabled by an interactive humanoid. Kerzel and Wermter (2017a,b) developed an end-to-end learning approach for object grasping based on a semi-autonomous self-learning cycle, which is described in more detail in section 4.1. Eppe et al. (2017) extended the approach with a modular, attention-based vision approach to grasp a diverse set of small objects in a cluttered scene. Kerzel et al. (2019a) further refined the approach by unifying the visuomotor architecture with a pyramidal convolutional network for identifying, localizing, and grasping a goal object in a complex scene.

#### 3.2.3. Datasets

A series of mono and crossmodal datasets from the above-described active sensing studies that have been recorded with NICO have been published for further use by the scientific community. Kerzel et al. (2019b) provide a haptic dataset of tactile and proprioceptive information during active haptic exploration of objects. Heinrich et al. (2019) provide a vision dataset of 60 object-hand interactions. Heinrich et al. (2018, 2020) recorded the EMIL dataset on embodied multi-modal interaction for language learning. The dataset focuses on low-level crossmodal perception during the environmental interactions from a body-rational perspective. The robot explored a set of toy objects through different actions like shoving, pulling, lifting, and scooting the object across the table. For each action, continuous recording from the robots' cameras, microphones, and proprioception, as well as from an external RGB and depth camera, is provided. Additionally, each sample is annotated with multiple natural language descriptions of the action.

#### 3.2.4. Human-Robot Interaction Evaluation

A wide range of HRI research questions has been addressed using the NICO platform. Initial studies focused on the reception of NICO and its emotional display. Churamani et al. (2017b) evaluated the seven abstracted facial expressions of NICO. Twenty participants (seven female, 13 male, aged between 19 and 49 years, English at a conversational level or better) from eleven different countries from Europe, Asia, South America, and the Middle East took part in the study. The participants could identify a subset of five expressions (neutral, happiness, sadness, surprise, and anger) with an accuracy of ≥75%. Furthermore, the effect of emotion-display on the subjective user rating was evaluated: users completed the Godspeed questionnaire (Bartneck et al., 2009) before and after having seen NICO's facial emotion display; the results showed a significant increase in ratings for the anthropomorphism, animacy, and likeability of the robot.

The use of emotion recognition and expression is aimed at the overall goal of creating natural and engaging interactions. This idea was further explored by personalizing the interaction with individual interaction partners and also by evaluating different “personalities” or interaction strategies for NICO.

In summary, these studies utilize NICO's ability for crossmodal sensing, motion, and social interaction to extend existing neurocognitive models, e.g., for emotion recognition, which were previously trained on prerecorded datasets (e.g., Barros et al., 2018), to live interactions. This allows the evaluation of the model's ability to adapt to individual users and, more importantly, it allows the effect of different HRI strategies on subjective user rating under realistic conditions to be studied. In the following section, we will further extend this research by evaluating how these more engaging interactions can benefit the learning of neurocognitive visuomotor models.

## 4. Methodology

In the presented HRI experiment, non-expert users perform a training procedure for a visuomotor task with the developmental humanoid robot NICO. Two conditions are compared: in the *robot-guided* condition, the robot takes an active role as a learner and guides the user through the process using an Embodied Dialogue System; in the *human-guided* condition a human experimenter gives all instructions to the participant and controls the robot. We evaluate both the effect on the subjective user rating of the robot and the effect on the learning process. The visuomotor learning task is based on an end-to-end approach for visuomotor learning by Kerzel and Wermter (2017b) and will be described in detail in section 4.1. To enable the bio-inspired development of grasping abilities from interaction with a physical environment, the robot repeatedly places and re-grasps an object on a table. This semi-autonomous learning cycle requires human assistance for initialization and also in the case of a failed re-grasping attempt.

### 4.1. Neural Architecture and Self-Learning Cycle for End-to-End Grasp Learning

**Figure 4:**
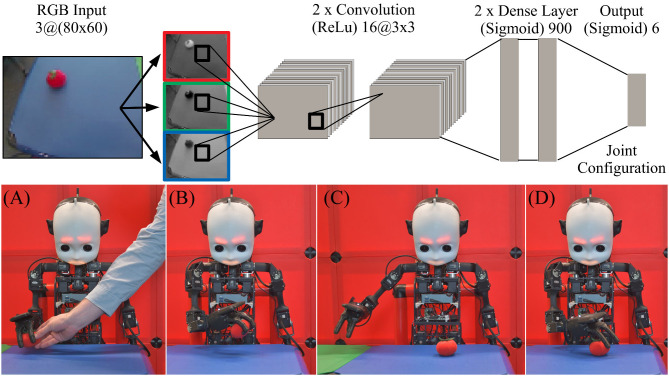
(Top) The neural architecture for grasp learning, adapted from Kerzel and Wermter (2017b), maps a visual input to a six-DoF joint configuration to reach for the object in NICO's field of view. (Bottom) NICO's self-learning cycle: **(A)** an object is put into NICO's hand. **(B)** NICO places the object at a random position on the table and records its joint configuration. **(C)** NICO releases the object, removes the hand, and records an image. **(D)** Using the recorded joint configuration, NICO re-grasps the object and repeats the self-learning cycle.

To circumvent the long and possibly damaging trial-and-error-learning periods required for reinforcement learning, Kerzel and Wermter (2017b) presented an approach for transforming the learning task into supervised learning with a neural architecture. A neural architecture can link a visual input image of an object in the robot's field of view to a joint configuration to reach for the object. This regression can be performed by a convolutional neural network (CNN). Figure 4 (top) shows the neural architecture. Given an input RGB image of 80 × 60 pixels, the two convolutional and two dense layers of the network predict a joint configuration for grasping. The two convolutional layers consist of 16 filters, each with a size of 3 × 3 and a ReLu activation function; the dense layers have 900 neurons each and, like the output layer with six neurons, one for each joint in NICO's arm, use a sigmoid activation function. The output is a joint position for reaching for the object, normalized to the interval [0, 1]. The architectural parameters were initially informed by successful approaches for learning visuomotor skills (e.g., Mnih et al., 2015), and empirically optimized (see Kerzel and Wermter, 2017a for details).

**Figure 5:**
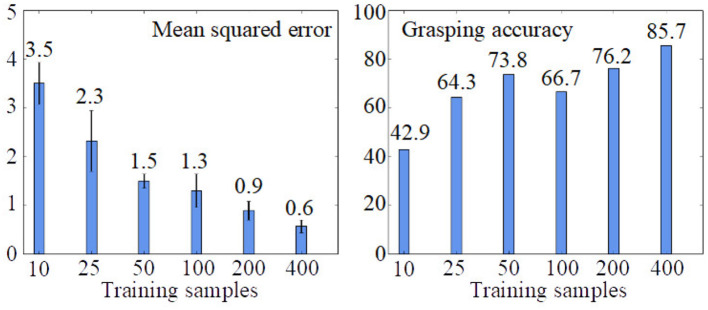
Results of the visuomotor learning from Kerzel and Wermter (2017b), based on the number of training samples, averaged over ten trials. With 400 training samples, 85.5% of all grasp trials are successful.

The neural architecture can associate an image of an object on the table with a joint configuration to reach for the object. For the supervised training of the network, annotated samples are needed that link said images to joint configurations. As it would be too time-consuming to manually create these samples, e.g., by guiding NICO's hand toward the object, the learning task was transformed: instead of grasping, NICO performs the far easier task of placing the object. Starting in an initial pose with the object in its hand, NICO places it at a random position on the table. It stores its current joint configuration, releases the object, removes the hand, and records an image. The resulting image-configuration pair will later be used to train the neural architecture. To complete the learning-cycle, NICO uses the stored joint configuration to re-grasp the object. Once the object is in its hand, NICO starts from the beginning and places the object at another random location on the table. Figure 5 (bottom) shows this semi-autonomous learning cycle.

**Figure 6:**
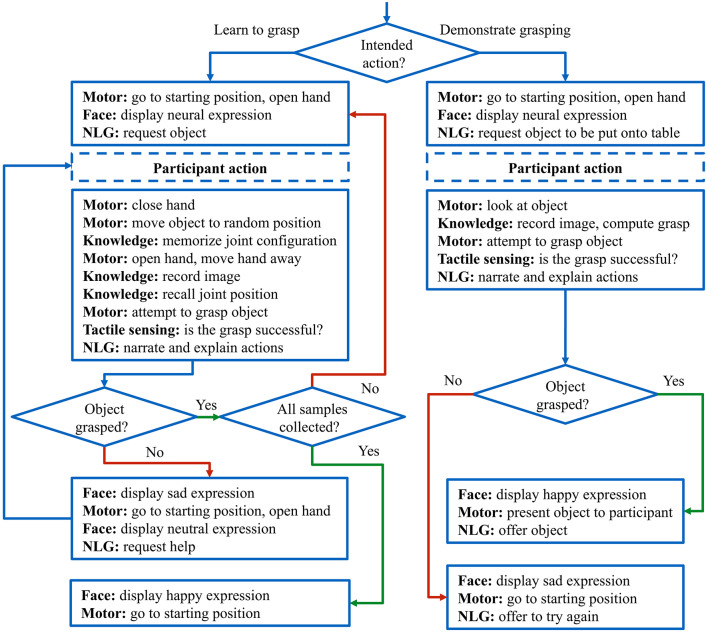
Diagram of the Embodied Dialogue System: depending on the task, NICO guides the user through the grasp training until a fixed amount of samples is successfully collected or demonstrates its grasp abilities to the participant.

Kerzel and Wermter (2017b) evaluated the architecture with training sets of different sizes (10, 25, 50, 100, 200, and 400 samples). All experiments were conducted for 2,000 epochs with stochastic gradient descent with Nesterov momentum (learning rate = 0.01, momentum = 0.9). The batch size was 40, except for the experiments with fewer samples, where a batch size of 10 for the 10-sample condition and a batch size of 20 for the 25-sample condition were used. Mean squared error was used as loss. Each condition was repeated ten times with Glorot uniform initialization and evaluated with 50 random test samples. Figure 6 shows results for different training set sizes. A grasp success rate of 85.7% was achieved with 400 samples of a single object. In a related study, Eppe et al. (2017) report an average accuracy of 76.4% using a total of 535 samples of six different objects. The later model was used for the demonstration phase during the HRI experiment. The success rates represent complete and successful physical grasp actions. A large portion of the non-successful grasps results from objects slipping from the robot's hand during the closure of the fingers or lifting of the hand.

However, two challenges arise: First, NICO needs to learn to place objects on the table. For this, initial motor training needs to be performed, during which NICO's hand is moved randomly over the table surface by a human assistant for a few seconds. Second, a human assistant is also required to place the training object into the robot's hand at the beginning of the learning process or when a re-grasping attempt fails. Using its proprioception, NICO can detect such a failure. It then stops the learning cycle, deletes the last collected sample, and moves back into its initial pose, waiting for a human assistant to place the object into its hand so that it can resume. The learning-cycle is semi-autonomous, as it can run for extended periods unattended. In the study by Kerzel and Wermter (2017b), errors occurred in about one out of thirty attempts; however, the number of consecutive error-free cycles fluctuated between 2 and 106. To allow human assistants to focus on other tasks while NICO is learning, the setup was further modified to not just halt the learning-cycle upon detection of failure but to also alert the experimenter, utilizing NICO's inbuilt communication abilities and the Embodied Dialogue System.

### 4.2. Embodied Dialogue System

The Embodied Dialogue System (Kerzel et al., 2017b) is designed as a control center connecting the six main components needed to accomplish visuomotor grasping tasks, namely Motion, Vision, Emotion, Computation, Knowledge, and Natural Language Generation (NLG). Motion controls the sensorimotor functions of the robot, while Vision uses the cameras in NICO's head to capture the stereo images necessary for the computation of joint values. The data for the task is stored and made available in the Knowledge component, while the Computation component handles the loading of the trained model to the neural network and the computation of the joint values for grasping. Communication with the user happens mainly through the Emotion component, which displays stylized facial expressions with embedded LED lights, and the Natural Language Generation (NLG), which outputs the situationally appropriate response or request through text-to-speech synthesis. The Embodied Dialogue System is implemented as an agenda-driven system, with the agenda being the training of object grasping skills or the testing and demonstration of the acquired ability. The Dialogue System implements the joint-task agenda approach (Piwek, 2017) in which tasks are accomplished via human-robot collaboration. NICO performs visuomotor actions and communicates its progress and the possible need for help, while the user hands the learning object to the robot and provides assistance when requested.

The Embodied Dialogue System is realized in a structured dialogue model (Schlangen, 2005) with atomic and finite states. Figure 6 shows the underlying state machine: states represent actions of the robot that are carried out with different combinations of components. The Embodied Dialogue System decides which action to perform next based on internal knowledge, external commands, or perception with visual or tactile sensors. If, for example, the *learning to grasp* task is selected, the system will initialize the learning process and ask for assistance from the user, it will execute the grasp-learning cycle until it reaches the desired number of successful samples or until NICO fails at a grasp attempt in which case assistance is required again. The NLG function is executed concurrently with other functions to report progress without interfering with the action currently being executed.

### 4.3. Experimental Design

#### 4.3.1. Participants

The recruitment of participants occurred through various sources (internet advertising, flyers, academic offices) to attract participants with no or little prior experience with humanoid robots, to avoid a so-called “convenience” sample (Baxter et al., 2016). The only requirement was a basic knowledge of conversational English. As an incentive, all participants were able to participate in a draw for gift certificates. Of the 24 participants (12 female and 12 male), an overwhelming majority (83.3%) reported no or little experience with humanoid robots prior to the experiment. Even though the participants were distributed randomly between the two conditions, the gender ratio remained equal in both. The overall average age of the participants was 27, with a range of 17–60. The majority (62.5%) identified as atheist or of no religion, 33.3% was Christian, and 4.16% as ‘other.' In terms of English proficiency, 45.83% self-assessed themselves as *advanced*, 41.6% as *intermediate*, and 12.5% as *beginner*. The study was approved by the Ethics Commission of the University of Hamburg. Written informed consent was acquired from every participant before the start of the experiment.

#### 4.3.2. Experimental Setup and Process

**Figure 7:**
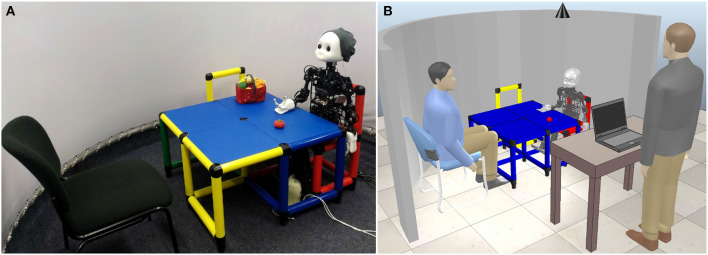
Experimental setup. Photo **(A)** and schematic depiction **(B)** of the experimental setup. The participant is seated face-to-face with NICO, while the experimenter is standing at a separate table and, depending on the experimental condition, guides the participant through the experiment and visibly operates the robot (*human-guided* condition) or remains silent (*robot-guided* condition). A ceiling-mounted camera records the interaction.

In the experimental setup, NICO is seated at a table with appropriate dimensions for a child-sized robot, as depicted in Figure 7. This experimental setup was initially introduced by Kerzel and Wermter (2017b) and subsequently adapted for various studies related to visuomotor learning and crossmodal object interaction (e.g., Eppe et al., 2017; Kerzel et al., 2017b, 2019b; Heinrich et al., 2018, 2020). Therefore, the experimental setup recreates a realistic neurorobotic learning scenario. The human participant is sitting face-to-face with the robot. The participant and the robot are enclosed by a semi-circular screen. A ceiling-mounted camera captures the interaction between participant and robot. The experimenter is positioned at an extra table to the side, where the interaction phase is started, observed and, depending on the experimental condition, narrated from. A separate adult-sized table was provided for filling out the questionnaires and the consent form.

**Figure 8:**
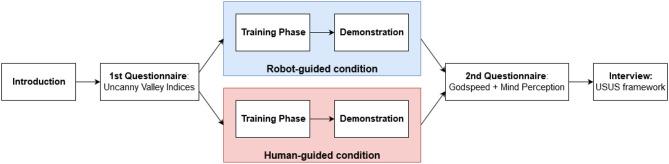
The experimental process. After an introduction, participants were asked to fill in a first questionnaire (Uncanny Valley Indices) based on their initial impression of NICO. Participants were then randomly assigned to the *robot-guided* or *human-guided* condition. In both conditions, participants performed a training phase with NICO, followed by a demonstration by NICO. After the demonstration, participants filled in two more questionnaires (Goodspeed and Mind Perception) and took part in a structured interview using the USUS framework.

Figure 8 shows the experimental process. To measure the effect of the Embodied Dialogue System on the effectiveness of the training process and the user's perception of the agent, independent measures were used. The participants were randomly assigned to either a *human-guided* condition (HG), in which the human experimenter guides them through the grasp-learning task, or a *robot-guided* condition (RG), in which NICO itself narrates the process and asks for help if needed. Before the experiment, a short introduction to the process and to NICO itself was given to the participants. The camera above the table was shown to them, and the purposes of both audio and video recording were explained. The participants had the opportunity to ask questions before written consent for their participation in the experiment was obtained.

Before the interaction, the participants were seated opposite an unmoving NICO and were asked to evaluate their immediate impression of NICO by filling out the first questionnaire, based on the refined version of the Uncanny Valley indices by Ho and MacDorman (2017), measuring perceived humanness, eeriness, separated into eerie and spine-tingling, and attractiveness. Since perceived humanness measures a similar concept to the anthropomorphism and animacy indices of the Godspeed questionnaires (Ho and MacDorman, 2010), this serves as a basis for the comparison of the change of the participants' impressions of the robot after the interaction.

The interaction was divided into two phases: A training phase and a demonstration phase. The training phase was limited to 10 min to allow a comparison of the number of collected samples in both conditions. To start the training process, the participant placed the object into NICO's hand. For an increase in interactivity, an intentional grasping error was included in the training. The error occurred randomly every two to five grasping attempts. (For comparison, without the intentional error, failed grasp attempts occurred on average only after more than 30 trials in the study by Kerzel and Wermter (2017b).

In the *human-guided* condition, the human experimenter instructed the participant when and how to initiate the training and narrated the process. NICO remained silent in this condition and only displayed facial expressions dependent on the success or failure of the grasping attempt. The human experimenter informed the participant if the robot was in need of assistance. In the *robot-guided* condition, NICO took over the role of the instructor, combining the display of emotions with verbal expressions of happiness or distress, and requesting the assistance of the participant if needed. The script for the training phase can be viewed in Table 1.

**Table 1:** Script for a training cycle in the first phase of the interaction.

**Visible robotic action**	**Human-guided dialogue**	**Robot-guided dialogue**
NICO moves into the starting position; NICO displays a neutral facial expression	*NICO is ready to train*.	*It's training time!*.
NICO opens its right hand	*Please put the object in NICO's right hand*.	*Please put the training object in my right hand*.
NICO places the object at a random position on the table	*NICO is choosing a location for the object*.	*Let's put the object here*.
NICO moves the hand away (and records a picture)	*NICO is remembering the location of the object*.	*I am remembering the location of the object*.
NICO attempts to grasp the object again	*NICO will now try to grasp the object*.	*I will try to grasp the object*.
**(SUCCESS)**		
NICO displays a happy facial expression; repeat from beginning	*NICO is choosing a new location for the object*.	*Let's put the object here*.
**(FAILURE)**		
NICO displays a sad facial expression	*NICO failed to grasp the object*.	*Oh, no! I failed to grasp the object*.
NICO moves back into starting position	*NICO deleted the last recorded file*.	*I deleted the last recorded file. I am done training*.

The demonstration phase consisted of the participant placing the object on the table in front of NICO three times, and the robot trying to pick it up. If the attempt was successful, the robot handed the object back to its interaction partner and, depending on the condition, voiced its happiness in addition to displaying an appropriate facial expression. The participants were given no instruction on where to place the object exactly. This allowed the participants to witness the effects of the prior training phase, even though a previously trained neural network was used. For the sake of transparency, the participants were informed about this fact beforehand. Equivalent to phase one, depending on the condition, either the human expert or the humanoid itself guided the participants through the process (see Table 2).

**Table 2:** Script for the grasping attempt in the second phase of the interaction.

**Visible robotic action**	**Human-guided dialogue**	**Robot-guided dialogue**
NICO moves into the starting position; NICO displays a neutral facial expression	*NICO is now ready to look, please put the object in front of NICO on the table*.	*Ready to look! Please put the grasp-learning object onto the table*.
No visible action (NICO records a picture)	*NICO is now looking at the object*.	*I am looking at the object*.
No visible action	*NICO is building the network and loading it from file*.	*Let me think about this very carefully. Building network. Loading network from file*.
No visible action	*The network is loaded and is now connecting to NICO*.	*Network loaded, connecting to myself*.
NICO reaches for the object	*NICO is now ready to grasp*.	*Output joint values. Ready to grasp*.
**(SUCCESS)**		
NICO grasps the object, lifts it up and presents it to the participant; NICO displays a happy facial expression	*NICO managed to grasp the object. Here you go, this is for you*.	*I grasped the object. Here you go, this is for you*.
**(FAILURE)**		
NICO does not grasp the object; NICO displays a sad facial expression and moves back into the starting position	*Since NICO failed to grasp the object, we will try again*.	*Oh, no! I failed to grasp the object. I will try again*.

After the interaction, the participants were asked to return to the interview table to fill in the second questionnaire, based on the Godspeed questionnaires by Bartneck et al. (2009) and the Mind Perception questionnaire by Gray et al. (2007), with some additional questions collecting demographic information about the participants. The Godspeed questionnaires measure anthropomorphism, animacy, likeability, perceived intelligence, and perceived safety. While they are known to be very dependent on the environment of the experiment and the experimental design (Weiss and Bartneck, 2015), more so than on the robot itself, they remain a popular evaluation tool and were included here for comparison's sake. The Mind Perception survey questions measure the amount of mind participants attribute to the evaluation subject, in two dimensions: Experience and Agency. While Experience is about how much the subject feels or senses, Agency describes the robot's capacity to act, plan, and exert self-control. As before, the participants were asked to evaluate NICO based on their personal impressions alone. The interview that followed was conducted in a semi-structured manner, with the questions based on the USUS evaluation framework of Weiss and Bartneck (2015), with a combination of obligatory and additional questions for the categories Usability, Social Acceptance, and User Experience. The interview was audio-recorded, about which the participants had been informed before the start of the experiment.

## 5. Results

### 5.1. Subjective Effect of an Active Role in Learning on the Participants

#### 5.1.1. Humanoid Evaluation

**Figure 9:**
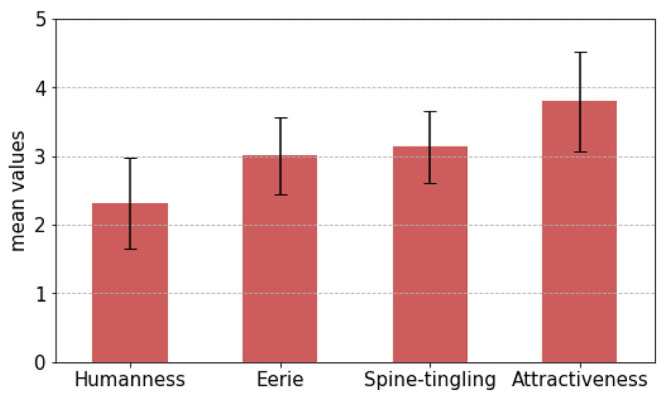
Initial evaluation to establish the participants' first impression of a silent, motionless NICO as a baseline for later comparison. Mean scores and standard error for the Uncanny Valley indices.

***Initial evaluation***. The Uncanny Valley indices were used to evaluate the participants' first impression of a silent, motionless NICO. The mean scores are *M* = 2.32(*SD* = 0.66) for humanness, *M* = 3.01(*SD* = 0.56) for eeriness, *M* = 3.13(*SD* = 0.53) for spine-tingling, and *M* = 3.79(*SD* = 0.72) for attractiveness. A visualization of the results can be viewed in Figure 9. These results establish a baseline against which we can make comparisons after the robot interaction in the two experimental conditions.

**Figure 10:**
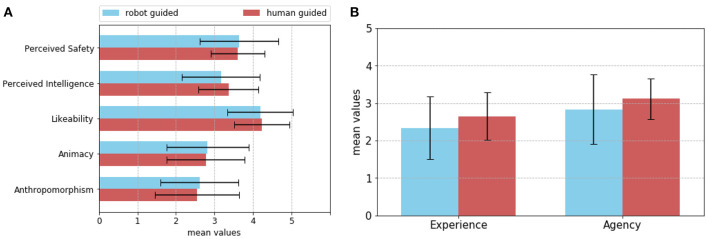
**(A)** Shows the mean scores and standard error for the categories of the Godspeed questionnaires. The active NICO scored higher in regard to Perceived Safety, Animacy, and Anthropomorphism. **(B)** Shows the mean scores and standard error for Experience and Agency in the Mind Perception survey questions. Blue signifies the *robot-guided* condition and red signifies the *human-guided* condition.

***Evaluation of the two experimental conditions***. As Figure 10 shows, the mean scores of the Godspeed questionnaires are slightly higher in the *robot-guided* condition for anthropomorphism, animacy, and perceived safety. In the *human-guided* condition, NICO ranked higher on likeability and perceived intelligence. The mean scores and standard deviations can be viewed in Table 3. To test the statistical significance of the different scores in both conditions, a two-sided Mann-Whitney test was performed. The evaluation of the differences between the two groups produced no significant results (*p*>0.05).

**Table 3:** Mean scores and standard deviations for the Godspeed questionnaires for both conditions.

	**Robot-guided**		**Human-guided**	
	**Mean**	***SD***	**Mean**	***SD***
Anthropomorphism	2.62	1.01	2.55	1.10
Animacy	2.82	1.07	2.78	1.01
Likeability	4.19	0.86	4.23	0.72
Perceived intelligence	3.17	1.01	3.37	0.78
Perceived safety	3.64	0.88	3.61	1.02

As for the results of the evaluation of the Mind Perception survey, NICO scored higher in regards to both Experience (*M* = 2.65, *SD* = 0.63) and Agency (*M* = 3.12, *SD* = 0.54) in the *human-guided* condition (Figure 10). The scores in the *robot-guided* condition were *M* = 2.34 (*SD* = 0.83) for Experience and *M* = 2.84 (*SD* = 0.93) for Agency. However, a two-sided Mann-Whitney test showed no statistical significance to the difference between the conditions (*p*>0.05).

***Evaluation after the interaction compared to the baseline***. To evaluate a possible change in perceived humanness during the interaction in relation to the established baseline, a Wilcoxon signed-rank test was performed, under the assumption that humanness measures the same concept as anthropomorphism, animacy, and likeability. As suggested by Ho and MacDorman (2010) in their analysis of the Godspeed questionnaires, a high correlation between anthropomorphism and animacy (*r*_*s*_ = 0.71, *p* < 0.01) and a medium correlation between animacy and likeability (*r*_*s*_ = 0.46, *p* = 0.03) was found. The small correlation between anthropomorphism and likeability (*r*_*s*_ = 0.35, *p* = 0.09) was not statistically significant.

The comparison of pre-interaction humanness and post-interaction anthropomorphism did not yield a significant result, with *p* = 0.11 (*Z* = 93.5). The *p*-value of the test within the *human-guided* condition is *p* = 0.7203 (*Z* = 34.5), and *p* = 0.11 (*Z* = 18.5) within the *robot-guided* condition. The comparison between humanness and animacy produced a significant result overall, with *p* < 0.01 (*Z* = 55.5), as well as within the *robot-guided* condition, with *p* < 0.05 (*Z* = 14). The test within the *human-guided* condition shows no significant difference (*p* = 0.17, *Z* = 21.5). The comparison between humanness and likeability produced a significant result overall, with *p* < 0.01 (*Z* = 4), as well as within the *robot-guided* condition, with *p* < 0.01 (*Z* = 2), and the *human-guided* condition, with *p* < 0.01 (*Z* = 0).

#### 5.1.2. Interaction Evaluation

The evaluation by interview reinforces and clarifies some of the conclusions of the statistical analysis; it also uncovers additional information by giving the participants the space to talk about their experience. The interview questions were based on the indicators of the USUS evaluation framework by Weiss and Bartneck (2015), and the interview was conducted in a semi-structured way. On average, the interviews took between 7 and 8 min per participant.

The effectiveness of the training process was perceived more favorably in the *robot-guided* condition, with a majority of participants believing in the accomplishment of a goal (60%) and experiencing a high level of satisfaction regarding NICO's progress (79.9%). In the *human-guided* condition, only 33.3% of the people reported a feeling of accomplishment, and only 16.6% were satisfied with the achieved performance.

All of the participants in the *robot-guided* condition rated the communication as satisfactory for them, and a majority (66.6%) felt that they would be confident enough to interact with NICO again in the future without the presence of an expert. The most commonly mentioned reason for their confidence was the fact that NICO had previously told them what to do. The remaining 33.4% who were unsure about future interactions mentioned insecurity regarding the expected extent of their help toward NICO. In the *human-guided* condition, the split was approximately even, with 58.3% of participants admitting the need for further help by a human assistant in a future interaction with NICO and 41.7% being confident in the simplicity of the task and in NICO's ability to conduct the training process without any mistakes. The majority of participants (60%) in the *robot-guided* condition reported a lack of anxiety due to the dialogue system and NICO's instruction. The remaining 40% expressed anxiety with regard to their own failings, but with the underlying theme being a concern for NICO and its learning process.

In the *robot-guided* condition, 73.3% of the participants felt that they played a more active role in the interaction, and 80% felt more integral to the success of the learning process. Meanwhile, people in the *human-guided* condition perceived themselves more frequently as passive observers or in a subordinate role, which is mirrored in the fact that they felt less important to the success of NICO's training. The Embodied Dialogue System also influenced how involved the participants felt in the whole interaction. Since NICO was not equipped with any additional functionalities that facilitate personal involvement, like face-tracking, the emotion display and dialogue system had to fill that role. As mirrored by the participants' perceived lack of importance to the training process in the *human-guided* condition, only 16.6% felt directly involved. Meanwhile, in the *robot-guided* condition, 73.3% felt a sense of personal involvement and continuous engagement, which was amplified by NICO addressing them and looking at them directly.

The interaction with NICO was perceived as enjoyable by both groups, with NICO's observable improvement and the feeling of teaching a child being the two most frequently mentioned reasons. But when asked about the type of roles NICO could fill in the future, dangerous, repetitive, or monotonous work featured more prominently in the answers of the participants in the *human-guided* condition. People in the *robot-guided* condition placed NICO mostly in elderly care or as a social companion robot. Additionally, 41.6.% of people in the *human-guided* condition cited NICO's lack of social interaction and emotional understanding as the main reasons for their refusal to accept NICO into their social circle. Meanwhile, 66.6% of people in the *robot-guided* condition could imagine welcoming NICO into their family, with the remaining 33.3% mentioning roles like “family pet” or “colleague at work.”

To summarize: the Embodied Dialogue System had a noticeable influence on the way people talked about and described NICO. In the *robot-guided* condition, user satisfaction was overall higher because the participants felt more integral to and engaged in the learning process. This was primarily attributed to NICO's vocal expressions, which made the participants overall confident enough to imagine a possible unsupervised interaction. In contrast, the people in the *human-guided* condition who experienced a higher level of confidence largely attributed it to the simplicity of the task. This overall detachment from the process and NICO itself can also be found in their active refusal to imagine NICO as more than a utility. This shows that in order to facilitate an environment that allows non-expert users to confidently supervise and actively assist the training process, the robot needs to be able to generate and keep the engagement of the user.

### 5.2. Objective Effect of an Active Role in Learning on the Learning Process

To ensure comparability between the two conditions, the first phase of the interaction was limited to a time frame of 10 min, during which the participant assisted NICO with the collection of samples. The number of collected samples during this training phase was on average higher in the *human-guided* condition (*M* = 9.08, *SD* = 2.63) than in the *robot-guided* condition (*M* = 6.5, *SD* = 2.53). This difference can, in large part, be attributed to the time the audio output of the dialogue system required.

However, in only the *robot-guided* condition, an interesting pattern could be observed: people were more inclined to engage themselves in the training process in a positive way, contributing to a smaller number of errors in the observed cases. By correcting the orientation and position of the object after NICO had placed it on the table, they made the subsequent repeated grasping less error-prone, leading to a larger number of uninterrupted iterations of the training process. This went as far as them actively putting the object back into NICO's hand after a predetermined failed grasp in the third or fifth iteration.

**Figure 11:**
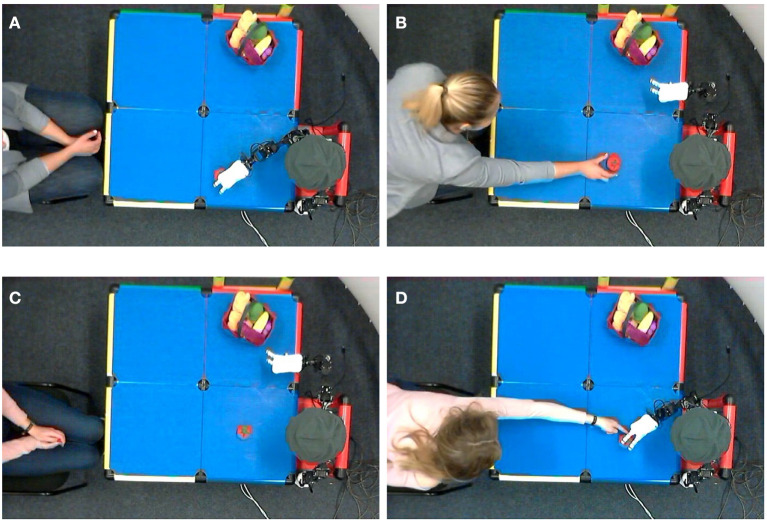
Participants correcting the object position during the training phase, after NICO put it on the table **(A,B)** and during a grasping attempt **(C,D)**.

In order to quantify this effect, the video recordings of the training phase were analyzed: out of 24 participants, 23 agreed to a video recording and a qualitative analysis of their interaction behavior. Therefore, 230 min of video were annotated for physical interactions between participant and NICO or participant and learning object. We defined any action in which the participant touches the training object or the robot as a physical interaction during the learning phase. Physical interactions were categorized as being either requested or participant-initiated. Depending on the experimental condition, the request for interaction could either come from the robot or from the experimenter. Interactions are requested for two reasons: first, to start the training process, the human participant is asked to put the training object into NICO's hand; second, if, during the learning process, NICO fails to grasp the object, which was artificially caused on each third to fifth trial, NICO moves back into its starting position, and the participant is asked to place the learning object into NICO's hand again. While there were no explicit reasons given for participant-initiated interactions, the fact remains that these interactions overwhelmingly occurred in the *robot-guided* condition, which can be linked back to the overall higher confidence in NICO's capabilities, as discussed in section 5.1.2, and a higher Perceived Safety score. The examination of the video material shows that participant-initiated interactions occurred if the robot lost its initial grip on the object before or while placing it on the table, in which case the participants picked it up and placed it where they assumed the robot had intended to place the object. The participants also corrected the position of the object after the robot released it, with a possible trigger for that interaction being that the participants observed the object moving during the release. The majority of participant-initiated interactions were small corrections to the object's position during an ongoing grasp attempt. It can be assumed that the participants predicted the end position of NICO's hand based on the observed trajectory and positioned the learning object accordingly. As shown in Figure 11, this also happened in physical contact with the robot^6^.

Table 4 shows the average requested and participant-initiated interactions per training phase. There is a visible increase in the number of participant-initiated interactions in the *robot-guided* condition (*M*_*R*_ = 1.08 compared to *M*_*H*_ = 0.18), even though half of them did not initiate any interactions at all. The prerequisites that could have enabled this behavior are discussed in section 5.1.2: participants have little fear of contact with the robot; they feel more engaged in the learning situation and more actively care about the learning outcome. The second observation, namely there being fewer requested interactions in the *robot-guided* condition (*M*_*R*_ = 2.67 compared to *M*_*H*_ = 2.91), can also be linked back to this. A possible interpretation of these combined results is that fewer requested interactions were necessary because the participants anticipated and prevented situations that would cause an unsuccessful grasp through participant-initiated interactions. While these results show no statistical significance for α = 0.05, there is a trend to be observed here: participants in the *robot-guided* condition showed more engagement and proactive behavior, which can have an effect on the actual neural learning processes of the robot.

**Table 4:** Requested and participant-initiated physical interactions during the learning phase, mean and standard deviations.

**Physical Interaction**	**Robot-guided**		**Human-guided**	
	**Mean**	***SD***	**Mean**	***SD***
Requested	2.67	0.98	2.91	1.30
Participant-initiated	1.08	1.83	0.18	0.40

## 6. Conclusion

### 6.1. Discussion of Results on Human-Robot Interaction

The participants reported a high rate of identification with active teaching or supporting roles and continued to refer to NICO as childlike. This indicates that the desired relationship dynamic of a teacher-learner team was achieved. The fact that the participants ascribed the reasons for their enjoyment to the feeling of teaching a child suggests that the collaborative learning approach further facilitated the student-teacher relationship dynamic. The results of the Godspeed questionnaires showed higher scores for anthropomorphism, animacy, and perceived safety in the *robot-guided* condition. Although the difference in ratings between the groups showed no statistical significance, the results of the interviews were able to confirm them to a degree. The participants attributed human characteristics to NICO in both conditions, but an examination of how they imagined NICO to react to their mistakes showed a tendency toward more human-like behavioral patterns in the *robot-guided* condition. Following the theory that a high anthropomorphism score could serve as an indicator of social acceptance, as suggested by Weiss and Bartneck (2015) in their meta-analysis of the Godspeed questionnaires, a greater disposition toward accepting NICO as part of their family or social circle could be observed among the participants in the *robot-guided* condition. Emotional support and elderly or health care appeared more frequently as imagined tasks for the active NICO in a domestic or work environment, which supports the higher anthropomorphism score.

Under the assumption, made in section 4.3.2, that humanness and anthropomorphism, animacy, and likeability measure a similar concept, a significant improvement of perceived humanness after the interaction with an active NICO could be observed. The effect was distinctly lower in the *human-guided* condition, suggesting that the Embodied Dialogue System was the influencing factor. Participants in the *robot-guided* condition reported a higher level of perceived involvement after interacting with NICO, although in both groups, the feeling of reciprocity peaked in the demonstration phase. This shows that the Embodied Dialogue System could help with keeping user involvement high throughout an interaction.

The number of collected samples and successful grasping attempts had no measurable influence on the perceived intelligence rating. A reason for this could be a missing basis of comparison for the users, amplified by the fact that the majority had no previous experience with humanoid robots. This is reflected in the fact that NICO's performance did not have any influence on the participants' sense of achievement. People in the *robot-guided* condition were on average more forgiving of NICO's mistakes, reporting an accomplished goal even with a low number of collected samples. In the *human-guided* condition, participants were much more ready to dismiss the learning process, even though a high number of samples indicated a fast training phase.

Although people in both conditions were equally afraid of making mistakes during NICO's training phase, the higher perceived safety score in the *robot-guided* condition indicates that participants overall felt more secure during this interaction. This is endorsed by the fact that the participants were more confident in their capability of interacting with NICO unsupervised, basing this confidence on NICO's clear instructions. This confidence is also mirrored in the fact that in the *robot-guided* condition, the participants more actively intervened in the grasp learning process by, e.g., correcting object positions (Figure 11). These results support the hypothesis that the Embodied Dialogue System enables non-expert users to supervise the learning process with confidence and efficiency.

### 6.2. Discussion of Results on Robotic Visuomotor Learning

The presented study shows that the Embodied Dialogue System can realize a successful visuomotor learning scenario between a non-expert user and NICO. Though the scenario was carried out in controlled laboratory conditions, the results indicate that the robot-guided learning interaction could also take place *ad hoc* in a domestic environment, e.g., when the robot encounters a novel visuomotor task and requires some form of human aid. This assistance can take different forms like demonstration (Gupta et al., 2016), advice, and instruction (Cruz et al., 2016) or physical assistance (Kerzel and Wermter, 2017b).

The positive subjective rating of the participants that was achieved in the *robot-guided* condition is an important factor for the use of humanoid robots as learning companions in everyday tasks. However, it is equally important to consider the quality of the learning outcome. Our results show that while using the Embodied Dialogue System to communicate its internal states and intentions took more time, resulting in less collected samples, it also led to a number of participants actively collaborating with NICO in its training and thus improving the learning process. The participants physically intervened in the learning process on their own initiative and prevented, e.g., unsuccessful grasps by correcting the position of the object. This behavior, in turn, enhances the quality of the collected samples. Moreover, in an actual learning scenario in the wild, learning based on the supervision of a human expert defeats the purpose of a semi-autonomous learning companion robot.

In summary, though fewer samples were collected in the *robot-guided* condition, the participants rated the *robot-guided* interaction more positively, indicating that they might be willing to spend more time teaching the robot, which could compensate for the slower speed of sample collection in this condition. Furthermore, participant-initiated interactions improved the quality of the collected samples. Finally, results from the interviews conducted indicate that the participants would put more trust in the abilities that result from NICO's training and are more willing to accept NICO as a companion in their home.

### 6.3. Discussion on Social Humanoid Robots

The presented study shows how a robot's ability to socially interact is a key factor for learning from and with humans. Considering the challenging nature of many real-world robotic tasks, the ability and willingness of non-expert users to aid in the necessary learning process is an important resource. The presented results are in line with the concept of developmental robotics (Cangelosi and Schlesinger, 2015): when looking at early human development, social interaction, especially scaffolding provided by caretakers, is critical for the development of cognitive abilities.

The results give evidence to support the proposition that successful scaffolding of a robotic learner in interaction with non-expert users is fostered by *sensory and motoric similarity, approachability, and social interaction abilities*. We show that this idea is reflected in the design of NICO (section 3.1) and by the previous studies carried out on NICO (section 3.2) evaluating it as a platform for social interaction, human-inspired active visual, auditory, and haptic perception, and developmental grasp-learning. The studies show in multiple cases that human strategies, e.g., active audio exploration (Eppe et al., 2018) and can be adapted to NICO. In turn, this also implies that non-expert users can apply their common sense and expertise as teachers in the NICO scenario. Multiple studies on Human-Robot Interaction demonstrate that NICO can engage in different Human-Robot Interaction scenarios and is rated positively by participants and that features like its facial emotion display have a positive effect on subjective user ratings (Churamani et al., 2017b). These properties of NICO are reflected in the questionnaire and interview responses of the presented study, and it can be assumed that they contributed to the positive learning outcome. The presented HRI study brings together, for the first time, human-robot interaction and visuomotor learning on NICO and shows that social interaction can be a key factor for enabling human teachers. The presented learning setup can be adapted to other platforms as a contribution to both the robotic machine learning as well as the developmental robotics community^7^.

### 6.4. Future Work

If a state-based Embodied Dialogue System is able to greatly improve the user experience of non-expert participants while teaching NICO, it might be possible to further amplify that behavior by designing a system that focuses on user comfort, not just to improve the experience of the human interacting with the robot but also to increase the training success of the robot learner. A natural learning process, with clearly communicated intentions, that is accessible to non-expert humans, will ultimately benefit both user and robot.

Valuable lessons can be drawn from the structured interviews for improving NICO's design and social interaction capabilities: NICO's three-fingered hands will be upgraded to four-fingered hands with a movable thumb, giving NICO a more human-like appearance. More importantly, the design will enable different types of grasps that can be selected according to object affordances. This more anthropomorphic design is intended to contribute to grasp learning with the aid of non-expert human teachers by enabling a more intuitive understanding of NICO's kinematics. To enhance NICO's overall appearance, NICO will be upgraded to allow concealed cable routing inside its limbs and also be fitted with an optional outer shell.

For the interaction scenario, suggestions by the participants will be implemented and evaluated: A module for face tracking and gaze shifts will be integrated into the API, as the missing eye-contact during the training phase was the most commonly mentioned grievance because it disconnected the user from the process. Also, a new text-to-speech module will be developed, as NICO's voice was also repeatedly remarked upon, either as being unfitting for a young child or causing confusion about NICO's perceived gender. At the moment, the evaluated Embodied Dialogue System only covers the instructions and assertions necessary for a smooth training process. A wish for a more detailed introduction to or narration of the process was mentioned, which could be a way to keep user involvement high even during longer phases without eye-contact and create a more satisfying user experience. In future studies, we will also more tightly control the participant's initiative in the interaction as an evaluation tool. With regard to crossmodal neurocognitive models, the suggested extensions will support a tighter integration of semi-autonomous reinforcement learning and multiple forms of learning from humans, like advice, physical aid, learning from demonstration, and human feedback as reward signal.

## Data Availability Statement

The datasets generated for this study are available on request to the corresponding author.

## Ethics Statement

The studies involving human participants were reviewed and approved by the Ethics Commission of the Department of Informatics of the Faculty of Mathematics, Informatics and Natural Sciences of Universität Hamburg. Written informed consent to participate in this study was provided by the participants' legal guardian/next of kin. Written informed consent was obtained from the individual(s) for the publication of any potentially identifiable images or data included in this article.

## Author Contributions

MK conceived the presented idea, developed the neurocognitive architectures and robotic learning setups used in the presented study. MK and TP-R designed the experiments, which were conducted and analyzed by TP-R. ES aided in the technical realization of the presented study, together with MK, one of the main developers of NICO. MK and TP-R were the primary contributors to the final version of the manuscript. SW supervised the project and revised the manuscript. All authors provided critical feedback and helped shape the research, analysis, and manuscript.

## Conflict of Interest

The authors declare that the research was conducted in the absence of any commercial or financial relationships that could be construed as a potential conflict of interest.

## Footnotes

^1^Visit http://nico.knowledge-technology.info for further information, the open NICO API, NICO CAD files, and released datasets.

^2^https://github.com/poppy-project/pypot

^3^https://www.pollen-robotics.com

^4^Further technical details, including CAD files for 3D-printed parts, a construction guide, video material, and the NICO API can be found at https://www.inf.uni-hamburg.de/en/inst/ab/wtm/research/neurobotics/nico.html.

^5^An overview video of selected studies carried out on NICO can be found at https://www2.informatik.uni-hamburg.de/wtm/videos/NICO_papers_2017-2020.mp4.

^6^Redundant safety features limit the closing force of the robotic hands: the mechanical design of the robotic hand is based on magnet connectors for the fingers that give away in case of excess load; furthermore, both software and firmware limitations for all motors are implemented. Therefore, the physical interaction between the participants and the robot was safe at all times.

^7^The NICO API and code examples, including a pre-trained neural grasping model, can be found at https://github.com/knowledgetechnologyuhh/NICO-software.
